# Highest Orthodontia

**Published:** 1905-07

**Authors:** 


### Reviews of Dental Literature.

HIGHEST ORTHODONTIA. 1. FACIAL BEAUTY; 2. DENTAL ANTAGONISM. By John Nutting Farrar, M.D., D.D.S., New York City.[1] (Continued from page 389.)

HARMONY BETWEEN THE DENTAL ARCHES AND FACIAL FEATURES.

In any of this class of operations, harmony between form and size of the dental arches, and that of the possible future form of the face of the child patient, is of utmost importance; this is necessary in order to accomplish the highest possibilities for the patient's best future personal interests, but this point will not be dwelt upon.

As perfect beauty is inherited or acquired, and all persons are susceptible to beauty, it is natural for all to desire to be as free from deformity as possible, therefore, between the utilitarian aspect (dental antagonism) and beauty of facial features, lies the object sought by all applicants.

Seldom do persons seek the dentist's services purely for the former (mastication of food), but they do often seek it purely for the latter (beauty). Therefore, in the treatment for correction of irregular teeth, the first aim should be to establish a change in their arrangement with the view to cause the highest improvement in the face, not only, as before said, in facial outline, but also in the intellectual expression, shown in and through the facial outline. As Bacon said, "The best part of beauty is that which no picture can express;" this part is the one to which I refer. Incongruity between a too broad dental arch in a narrow face is no less a defect than a too narrow arch in a broad face. Both cause a weakness of intellectual expression. Unless there is harmony between the

---

[1] Lecture delivered before the New York Dental College Alumni, January 18, 1905.

dental arches and the size of the face, as a whole, the accomplishment simply of perfect antagonism cannot be regarded all that is for the patient's good. To so regulate teeth that the moulding will bring out the intellectual scintillations through the smiling wrinkles in the lower half of the face, balancing it with the wrinkles about the eyes, is high art. The former of these results (perfect antagonism) may be accomplished without the latter (beauty). To kill out the intellectual fire by causing a blank face, often gross, and an expression of "all teeth" with the cuspids standing too far apart, stretching the skin so as to kill the finer emotional expressions about the lips, is not evidence of artistic skill.

Harmony between the arrangement of the teeth and the facial features may call for evenly arranged teeth, or it may call for slightly irregular teeth, depending upon the form or contour of the face and head. If the patient has that which is called "high cheek bones" and an aquiline nose, with a corresponding mentality, the teeth should be slightly irregular in order to harmonize. A selfish expression, however, may not necessarily be intensified by inclining the teeth posteriorly, like those of a serpent, nor by leaving the cuspids so long as to cause a ferocious expression. If any deviation is allowed to be made, harshness of expression may be softened somewhat, but not to the extent of causing too much incongruity between the intellectual and the physical, for that would cause an expression of deception. One of the most important aims should be to keep the upper cuspids down as close to the lower teeth as is prudent for proper occlusion of the arches, and not have the six anterior teeth appear like a straight line.

As before implied, to accomplish the highest degree of beauty, and at the same time accomplish perfect antagonism, is not always possible. While perfect antagonism is of great importance, it should not, as elsewhere mentioned, be made at the expense of beauty of facial outline and intellectual expression. To repeat: Every regulator should have high ideas, and always aim upward; high art in the correction of deformities should be the main aim, and should be carried out, as far as possible, but of course no dentist should expect these excellencies in results who does not have some natural artistic gifts and clear knowledge of the ideals known to great artists concerning the proportion of different parts of the human face, and, as before said, know the comparative merits of the several different classes of faces sufficiently to determine at a glance the points that can and the points that cannot be improved by moving the teeth. But to know well the degree to which this knowledge can be best applied in a case, and the degree that circumstances of the patient's health, social position, educational and business environments will permit, must indeed be evidences of sound judgment.

The meaning of the term beauty, of course, may differ with different persons, but the essayist means, by the term beauty, a physical and intellectual harmony of expression that everybody loves to see. In some cases it is not only possible to accomplish this balance, but to stimulate personal confidence, so that it (confidence) will cause an expression of self-reliance that will strengthen and cause an appearance of a balanced mind, free from self-mortification, sometimes engendered by consciousness of personal deformity.

Whether it is right to carry facial beauty in appearance beyond the power of the brain may be questionable, but the essayist thinks that less harm is caused by so doing than by the reverse results, especially in women. The only injury, sometimes noticed, is stimulation of personal pride, into temporary degeneracy called vanity.

THE PHILOSOPHY OF THE AUTHOR'S PLAN VS. THE OLD PLAN.

There are many cases in which the upper arch requires more or less widening in order to establish proper antagonism, but to widen the upper arch beyond the accomplishment of proper antagonism can hardly be regarded as scientific. I shall not attempt, in the limited time here given, to go over the entire field of this question, but shall confine my remarks to the main principles.

In cases of narrow arches, caused by mouth-breathing or by wrong antagonism, or little or no antagonism, it is generally the upper arch that is most at fault. In these cases, the arch tends to narrow more than does the lower. Indeed the lower seldom shows this fault. This is because the position of the upper teeth, by failing to antagonize with the lower teeth (which when properly arranged has greatly to do with keeping the upper in their proper places), go astray by having too much liberty and by pressure of the cheek muscles against them while in the act of swallowing.

The upper teeth depend more upon antagonism with the lower teeth than do the lower depend upon antagonism with the upper. When the upper teeth are thus properly regulated, and the lower are not moved, permanency of the upper teeth is much more easily secured than when the lower teeth also are moved, which leaves them more or less loose. The compactness of the teeth that contribute to make up the lower arch is greater than that which contributes to the maintenance of the upper arch. If a side tooth in the lower arch is lost, this defect in the strength of the arch is proved by the inclining forward of the tooth in rear of the space left by the lost tooth. If a side tooth in the upper arch is lost, the tooth posterior to the space does not tend to incline nearly so far, under similar conditions, as in the lower arch. The tooth anterior to the space may drift posteriorly, but if it does drift, it is generally only slightly.

The stone arch principle is greater in the lower than in the upper arch. In the well-formed lower, all the teeth may be regarded as buttress keys of the arch, while in the upper, even if well formed, the key principle in most cases is weak or nearly wanting. In early adult and in middle life, the upper teeth are mainly held in place by the lower teeth, aided by the cheek muscles. If properly arranged, the lower teeth not only prevent the upper arch from being strongly self-binding, but the (upper) arch needs no key. The teeth of the upper arch, besides being held by the cheek muscles, are prevented from narrowing by the overhanging of the cusps of the lower teeth. Firm upper teeth are held in place by antagonism with the lower teeth. If the lower teeth are loose the antagonism of the upper may or may not be made firmer. If in either arch, however, there are spaces caused by loss of some of the teeth, and the remainder do not lock properly while in antagonism, more or less of them may go astray. As we all know, there are two kinds of antagonism,—cutting antagonism and crushing antagonism. The six front teeth are for the former, while the teeth in the rear of these six are for the latter purpose.

There are two distinct teachings in regard to the treatment of protruding teeth, and also for irregular anterior teeth; both claim improvement through the theory of antagonism. The essayist's plan (since 1864) is founded upon that which he regards as the broad basis of æsthetics, which embraces also efficient antagonism. The other plan is simply to even the teeth of one jaw without much regard (excepting in name) to antagonizing with the teeth of the opposite jaw, and with slight, if any, regard to æsthetics, or the effect of the operation upon the facial features. One is permanent regulation of the teeth of both jaws; the other, the doubtful and almost certain destruction not only of lasting proper antagonism, but injury to facial beauty.

One plan has for its object, first, to beautify the face; second, the accomplishment of permanent efficient antagonism, not necessarily perfect antagonism in all cases, that is firm and strong and not liable to go astray by antagonism. The other plan is to widen the upper arch until all the irregular upper teeth are brought into line, and then leave them, or else widen the lower arch to match, a plan that leaves both arches with little or no support, irresponsible and liable to destroy the equilibrium unless held by artificial retainers.

EXTRACTION.

The treatment of cases of jumbled front teeth or protruding teeth by the essayist's plan, when it is not necessary to widen the upper arch, is to shorten it by extraction of a bicuspid on one, and possibly on both sides, and then to move the teeth anterior to the space posteriorly or diagonally back, along the dental line, and not attempt to widen the arch. The other operation is to shorten the protruding part of the arch by widening it, even if it injures facial expression. By the essayist's plan the operation is not only easier and shorter, but firmness of antagonism is not at all disturbed, and facial expression is improved, not weakened. To extract a first bicuspid and then move the cuspid into the space made, leaves the cuspid in the place of the outer cusps of the extracted first bicuspid. To move the lateral incisor into the place of the cuspid, following this by moving the central, does not disturb antagonism, nor does it in any way injure facial expression, for the lacking of one bicuspid is not noticeable when the line of the upper teeth are properly closed up, because the arch, while it is shortened, leaves none of the weaknesses between the two arches that is caused by over-widening. This plan is natural antagonism, the other is loose artificial antagonism; this one is firm, strong, reliable, generally intensifying facial beauty, while the other scatters and weakens it.

Sometimes it is better to extract a second bicuspid instead of the first, but this depends upon the extent of irregularity to be corrected in the anterior part of the arch.[1]

---

[1] This point may be found explained in Farrar's "Irregularities of the Teeth," vol. i.

